# A Diverse *Vibrio* Community in Ría de Vigo (Northwestern Spain)

**DOI:** 10.3390/biology13120986

**Published:** 2024-11-28

**Authors:** Xiaoyun Huang, Keyi Huang, Sihan Chen, Xinglan Yin, María Pérez-Lorenzo, Eva Teira, Emilio Fernández, Xiaolei Wang

**Affiliations:** 1 College of Marine Life Sciences, Frontiers Science Center for Deep Ocean Multispheres and Earth System, Ocean University of China, Qingdao 266003, China; hxy764379760@163.com (X.H.); huangkeyi226@163.com (K.H.); cshkkk2022@163.com (S.C.); yyjjkk0628@163.com (X.Y.); 2 Institute of Evolution & Marine Biodiversity, Ocean University of China, Qingdao 266100, China; 3 Centro de Investigación Mariña da Universidade de Vigo, Departamento de Ecoloxía e Bioloxía Animal, Facultade de Ciencias do Mar, Universidade de Vigo, 36310 Vigo, Galicia, Spain; mplorenzo@uvigo.es (M.P.-L.); teira@uvigo.gal (E.T.)

**Keywords:** *Vibrio* community, diversity, Ría de Vigo, aquatic ecosystem, aquaculture

## Abstract

The genus *Vibrio* is genetically and ecologically diverse, resulting in severe economic losses in aquacultural macroalgae and animals. Studies on vibrios will contribute to the conservation of aquatic aquaculture in Ría de Vigo (Spain), which is famous for its shellfish farming activity. However, limited research focused on the diversity and distribution of *Vibrio* spp. in Ría de Vigo has been reported. Here, a slight increase in the abundance of *Vibrio* spp. was recorded from inshore to the open sea by quantitative PCR (qPCR). Vibrios were more abundant in the free-living (FL) than in the particle-associated (PA) fraction, showing that FL might be their preferential lifestyle. *Photobacterium piscicola*, *Vibrio japonicus* and *Vibrio harveyi*, which are serious pathogens for fish and invertebrates in mariculture, were found to be the dominant species across all samples by high-throughput sequencing and analyses and were mainly affected by stochastic processes. More colony-forming units and species of vibrios were recorded at 16 °C (18 species) than at 28 °C (6 species), and five isolates (5.05% of the total isolates) showed low 16S rRNA gene similarity (<98.65%) to the known strains. The results indicate that culture conditions closer to the in situ temperature may help to isolate more *Vibrio* species. Our findings may contribute to local aquatic aquaculture research, providing background information and novel insights for management and pathogen prevention in aquaculture, and more studies should focus on Ría de Vigo to acquire novel or pathogenic microbes in the future.

## Simple Summary

Many *Vibrio* species can be pathogens for aquatic macroalgae (e.g., *Saccharina*), animals (e.g., fish and shellfish) and even humans. Studies on the diversity and ecological distribution of *Vibrio* spp. will contribute to the conservation of aquatic aquaculture and have extremely important research value in terms of economic developments. Therefore, in this study, the distribution patterns of total and culturable *Vibrio* spp. in seawater along Ría de Vigo were investigated by cultivable methods, qPCR and high-throughput sequencing. The results of this study can provide a reference for management and pathogen prevention in aquaculture ecosystems in the context of global climate change.

## 1. Introduction

The genus *Vibrio*, one of the biggest genera within the class *Gammaproteobacteria*, is a group of ubiquitously distributed marine heterotrophic bacteria within which more than 150 valid species belonging to the genus *Vibrio* (https://lpsn.dsmz.de/genus/vibrio, accessed on 10 November 2024) have been reported [1,2]. They frequently occur in close association with marine plants and animals, where they can act as mutualistic symbionts [3]. As famous opportunistic pathogens, several *Vibrio* species can cause infections in humans [4], marine animals (e.g., fish and shellfish) [5] and algae (e.g., *Saccharina*) [6]. For example, *Vibrio cholerae* can cause cholera, a severe diarrheal disease that can be quickly fatal if untreated and is typically transmitted via contaminated water and person-to-person contact [4]. About 200 recognized O serogroups are known, and only serogroups O1 and O139 have been found to be associated with severe disease and cholera pandemics [7]. *Vibrio harveyi* is a well-recognized and serious bacterial pathogen of marine fish (e.g., *Acanthopagrus cuvieri* and *Epinephelus tauvina*), leading to eye lesions/blindness, gastro-enteritis, muscle necrosis, skin ulcers and tail rot disease [5,8,9]. Diseased fish and even fish-excreted microplastics may further become considerable vectors of pathogenic vibrios to surrounding organisms, increasing the risks of *Vibrio* infection in aquatic ecosystems [10]. Additionally, *Vibrio* species (e.g., *Vibrio mediterranei*) have been identified as the pathogenic bacteria responsible for rotten hole disease in *Laminaria japonica* (synonymous with *Saccharina japonica*) and yellow spot disease in *Pyropia* [6,11]. Consequently, research on marine *Vibrios* will contribute to pathogen prevention in aquaculture.

The *Vibrio* population generally represents ~1% of the total bacterioplankton in most sea areas, indicating that it is a low-abundance constituent in microbial assemblages [2,12,13]. However, the *Vibrio* metabolism is fast, and vibrios have short generation times (~10 min) and can rapidly increase in number, becoming dominant groups after phytoplankton “bloom” events (e.g., diatoms and *Phaeocystis*) and micronutrient inputs (e.g., iron), as well as in response to pulses of dissolved organic nutrients (e.g., glucose and amino acids) [14,15]. Due to their low abundance and the active or dormant microbial taxa present in an environment at a given time, vibrios can be seen as rare taxa [16]. In recent years, the significance of rare biospheres has gained attention [17]. Rare biospheres usually contribute significantly to both α- and β-diversities of whole communities and constantly play critical ecological roles in specific ecosystems [18]. High phylogenetic and functional diversities provide ecosystems with a form of “insurance” or a buffering capacity against environmental changes [18]. Additionally, vibrios may consume a wide range of marine organic matter, including chitin, alginate and agar, derived from marine algae and animals [13,16]. For example, several *Vibrio* species have been reported to contain chitinases and are thus able to use chitin—a polysaccharide very abundant in the ocean—as a carbon source [13]. Thus, vibrios have been considered to play important roles in marine ecosystems, especially in marginal seas and estuaries [2,12].

*Vibrio* spp. is widely distributed in marine environments, from coastal areas to deep-sea hydrothermal vents and the Mariana Trench [16,19,20]. *Vibrio* communities can generally be categorized into free-living (FL) and particle-associated (PA) communities. When adopting the FL strategy, *Vibrio* needs to swim in the water column via polar flagella to sense and acquire nutrients, whereas particle-associated communities need to colonize particles and/or living hosts to acquire nutrients or to avoid predators [21]. The abundance and community structures of *Vibrio* spp. vary greatly in different sea areas so they can adapt to various environments. Higher *Vibrio* abundance has been recorded in seawater and sediments of the Chinese marginal seas than in other areas [16]. *Vibrio* sp. OTU13800 and *Vibrio mimicus* are the dominant groups in the Sydney Harbor estuary [1], and *Vibrio atlanticus* and *Vibrio owensii* are the dominant and active species in the Changjiang estuary [12]. In the Indian Ocean, *Vibrio rotiferianus* is the dominant species [22], and different species are found in high abundance throughout the Chinese marginal seas [3,16]. Unstudied sea areas like Ría de Vigo (Atlantic Ocean) may harbor different *Vibrio* species because of their local environments.

Many reports have attempted to explain the main factors determining *Vibrio* dynamics under various environments, finding that temperature and salinity are considered the most common key factors affecting marine vibrios [3,12,16]. Other environmental parameters, including dissolved organic carbon (DOC), dissolved oxygen (DO), nitrogen, phosphate, pH, chlorophyll *a* and turbidity, often explain inconsistent variance in *Vibrio* spp. [16]. It has been proven that DOC has strong effects on the distribution of *Vibrio* spp. [16]. In a microcosm incubation experiment involving *Ulva prolifera*-derived DOM, vibrios robustly and rapidly grew over short timescales (6–24 h), with their relative abundance accounting for up to 52.5% of active bacteria [23]. In nutrient-enriched microcosms containing glucose, nitrate and methylphosphonate, substrate-driven microbial succession was observed, with vibrios blooming rapidly in response to nutrient addition [24]. However, due to its various organic components, the effect of DOC is multifactorial and should be taken seriously in future research [13]. Additionally, stochastic processes have received considerable attention in recent years, suggesting that the microbial community assembly may be affected by other events (e.g., reproduction, death and dispersal) [25]. Stochastic processes have been reported to govern the turnover of marine *Vibrio* communities in the Beibu Gulf (China) and play important roles in shaping the geographical pattern of the vibrionic community in the Yellow Sea (YS), East China Sea (ECS) and South China Sea (SCS) [16]. To fully understand how biotic or abiotic factors affect *Vibrio* dynamics, investigations across different oceanic regions would be needed.

Ría de Vigo is an incised valley originating from the combined action of tectonic and erosive processes and is located at the northern border of the Northwestern (NW) Iberia–Eastern Africa boundary upwelling ecosystem [26]. It is characterized by a temperate oceanic climate with prevailing north and southwest winds. The depth of Ria de Vigo ranges from 7 m in the inner part to 45 m at the outer southwest sea entrance, comprising diverse sedimentological conditions [27]. Upwelling events promote a quick renewal of the water volume replaced by cold, oxygenated and nutrient-rich subsurface oceanic water, which translates into high primary production rates [26]. This high productivity allows for highly productive shellfish farming activity, which produces up to 37,000 tons of mussels annually [28]. The Ria is impacted by diverse anthropogenic activities, such as increased sedimentation rates and elevated levels of certain heavy metals [29,30]. The input of partially treated urban sewage water into the water column is also likely to be an important environmental problem [28]. Thus, it is a perfect example of the coexistence of human populations and the consequent alteration of the marine ecosystem [26], making it a suitable model system to explore the ecological distribution of eutrophic microorganisms (especially *Vibrio*) and their environmental responses.

In this study, we investigated the dynamics and environmental drivers of both total and culturable *Vibrio* spp. along Ría de Vigo (Spain). Culturing, quantitative polymerase chain reaction (qPCR) and amplicon sequencing were conducted to study the distribution patterns of *Vibrio* spp. and identify potentially novel species. We hypothesize that there is a diverse *Vibrio* community in Ría de Vigo which may affect shellfish and fish farming activities.

## 2. Materials and Methods

### 2.1. Sampling

**Figure 1:**
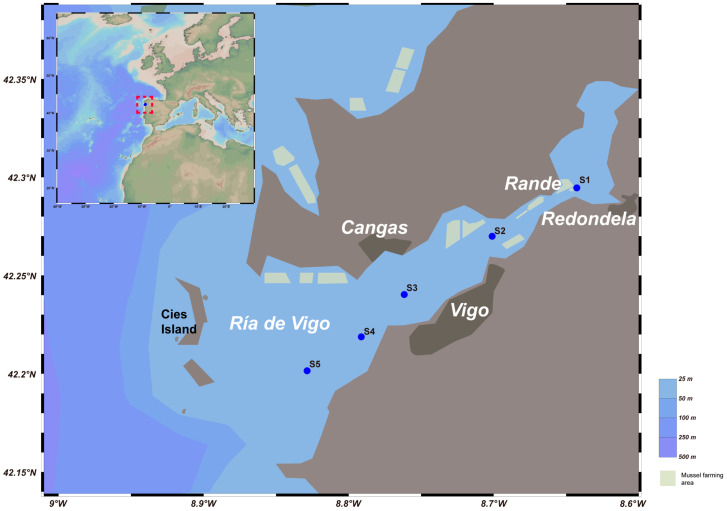
Map showing locations of study area and sampling sites. Map was created using Ocean Data View (version 5.5.2; R. Schlitzer, Ocean Data View, https://odv.awi.de, accessed on 11 July 2021), and mussel farming area was added [33]. Blue plots represent sampling sites S1–S5. Details are shown in Table S1.

Ría de Vigo has a large human population, and highly productive shellfish farming activity occurs in this area, making it an ideal area to study the response of eutrophic vibrios to local environments. A total of twenty samples (with two depths and two filter sizes at each site) were collected from 5 sampling stations located in Ría de Vigo from inshore to the open sea in 11 October 2017 (Figure 1). Surface seawater (SW) and bottom seawater (BW) samples were collected at each station using Niskin bottles. Vertical profiles of temperature, salinity, dissolved oxygen and fluorescence were obtained with an SBE25 CTD. From each sample, 1 L of seawater was filtered in sequence through 3 μm (TSTP, 47 mm) and 0.22 μm (GTTP, 47 mm) polycarbonate membranes (Millipore Corporation, Billerica, MA, USA). Microorganisms on the 3 μm filter were considered bacteria associated with smaller particles and algae (PA), whereas microbes on the 0.22 μm filter were considered free-living bacteria in seawater (FL) [31]. Subsequently, all filters were stored in liquid nitrogen on board and transferred to a −80 °C freezer for preservation in the laboratory. Samples for the Chl *a* analysis were collected using 0.7 μm GF/F filters (Whatman, Maidstone, UK). Chl *a* was extracted with 90% acetone for 24 h in the dark and determined using a Turner Designs Trilogy Laboratory^®^ Fluorometer (Turner Designs, Sunnyvale, CA, USA) [32]. The stations sampled in this study are shown in Figure 1, and detailed station information and sample information about each sample are shown in Table S1.

### 2.2. Isolation of Culturable Vibrios

In triplicate, 200 μL of each water sample and dilution was spread onto ZoBell’s 2216E agar (MA; Becton, Dickinson, Detroit, MI, USA; 10^−3^–10^−5^ in dilution) and thiosulfate–citrate–bile salts–sucrose (TCBS) agar plates (HopeBiol, Qingdao, China; 10^−1^ in dilution). After incubation at 28 °C (usually used for the isolation of marine bacteria) and 16 °C (near in vitro environments) for 48 h, the number of colony-forming units (CFUs) was counted. Single colonies were randomly picked and purified on 2216E medium under 28 °C and 16 °C, respectively. The genomic DNAs of fresh bacterial cultures were extracted using a boiling method [12]. Using genomic DNA as the template, primers (B8F and B1510) were used to amplify the pure strains. PCR reaction system and cycling conditions were used according to Liu et al. [34], and the amplified 16S rRNA gene sequences were sequenced at Beijing Genomics Institute (BGI, Qingdao, China). Taxonomic identifications of bacteria were assigned in the EzBioCloud database (https://www.ezbiocloud.net/; accessed on 23 August 2023) and GenBank database (https://blast.ncbi.nlm.nih.gov/Blast.cgi; accessed on 24 September 2024) based on the 16S rRNA genes. For the potentially novel species, PCR amplification, cloning and sequencing of the 16S rRNA genes were performed according to a previous report [35]. The almost complete 16S rRNA gene sequence (~1500 bp) was manually checked and submitted to the GenBank database. Pairwise sequence similarity values between potentially novel species and closely related type strains were calculated using the EzBioCloud server [36]. All of the pure strains were stored at −80 °C with 15% (*v*/*v*) sterilized glycerol.

### 2.3. DNA Extraction and Quantitative PCR (qPCR)

The abundances of total bacteria and *Vibrio* spp. were tracked using qPCR technology with SYBR-green detection based on the 16S rRNA genes. According to the methods used in a previous report [12], the genomic DNA of the environmental samples was extracted using a DNeasy PowerSoil kit (QIAGEN, Hilden, Germany) following the manufacturer’s instructions. The extracted DNA was resuspended in 50 μL of TE buffer (1 M Tris–HCl, 0.5 M EDTA, pH 8.0) and stored at −80 °C. qPCR was performed using the StepOnePlus^TM^ Real-Time PCR system (Applied Biosystems, Foster City, CA, USA) and StepOne software (version 2.2). V567F and V680R were used as specific 16S rRNA oligonucleotide primers for vibrionic qPCR reactions, whereas B967F and B1046R (specific primers for total bacteria) were used to quantify the total bacterial taxa. The primer sequences, reaction mixture and cycling conditions were created based on the descriptions outlined by Wang et al. and Zhao et al. [12,37]. The standard was prepared using the 16S rRNA gene of *Vibrio rotiferianus* WXL191 (our laboratory) following the methods used in our previous work [37]. All extracted environmental DNA was diluted 5 times to reduce the absorption error, qPCR was carried out in triplicate and all amplification efficiencies were between 95 and 105% with R^2^ values > 0.99.

### 2.4. High-Throughput Sequencing and Processing

To determine the community composition of *Vibrio* spp. at each site, the *Vibrio*-specific 16S rRNA gene primers V169F and V680R were used for amplicon sequencing. The reaction mixture and cycling conditions followed the descriptions of Wang et al. [12]. After confirming positive amplicons by agarose gel electrophoresis, the PCR products were purified using 2% agarose gel and sequenced on the Illumina Miseq PE300 platform at Majorbio Bio-Pharm Technology Co., Ltd. (Shanghai, China). To remove the short-length sequences (<100 bp) and low-quality sequences (<20), raw reads were trimmed with FASTP [38]. Using FLASH with at least a 10 bp overlap and <5% mismatches, paired-end DNA sequences were joined [39]. Chimeric sequences, barcode and primer sequences were removed by the DADA2 plug-in within the QIIME2 software (https://qiime2.org/) [40,41]. Vibrionic sequences were clustered into operational taxonomic units (OTUs) using the QIIME2 software at a 97% sequence similarity level. The taxonomy of representative sequences for each OTU was assigned by local blast against the SILVA v138 database (a minimum support threshold of 70%) and reassigned against the EzBioCloud database (https://www.ezbiocloud.net/; accessed on 23 August 2023) to obtain a more accurate taxonomic result.

### 2.5. Statistical Analyses

To minimize biases associated with sequencing coverage, the number of sequences for each sample was homogenized to the lowest number of reads (28,220 reads) by a script in R software (version 4.4.2) [37]. Alpha (α)-diversity was described by Shannon, Simpson, Ace, Sobs, Pielou, Chao 1 and Simpson even indices, which were calculated using the “vegan” package. The differences between the FL and PA groups were examined using a *t*-test. For β-diversity, a principal coordinate analysis (PCoA) was performed at the OTU level using the “vegan” package. The subsequent analysis of similarities (ANOSIM) was performed using the anosim function with 999 permutations in the “vegan” package. The distance–decay pattern for *Vibrio* spp. was conducted by using the “vegdist” function (“vegan” package version 2.5-7). Variation partition analysis (VPA) was performed using the “vegan” package to estimate the relative contributions of geographic distance and environmental factors to the *Vibrio* community structure. Distance-based redundancy analysis (db-RDA) was used to assess the effect of measured environmental factors on PA and FL microbes using “vegan” package. To reveal the relationship between environmental factors and microbial communities, Mantel test based on Spearman’s correlations was carried out using the “ggcor” package (version 0.9.8.1). To determine the relationship between *Vibrio* species and environmental factors, the IBM SPSS Statistica (v 23.0.) software was operated to calculate Spearman’s rank correlation coefficients. Linear regression analyses between α-diversities, abundance and salinity were calculated using the “lm” function and visualized using ggplot2. Additionally, a neutral community model was created to estimate the effects of stochastic processes on *Vibrio* community assembly. Model computations were performed with a custom R code according to previous reports [42]. The community assembly was more consistent with stochastic processes when R^2^ was close to 1, whereas assembly was more affected by deterministic processes when R^2^ was close to 0 [43].

## 3. Results

### 3.1. Environmental Conditions

**Figure 2:**
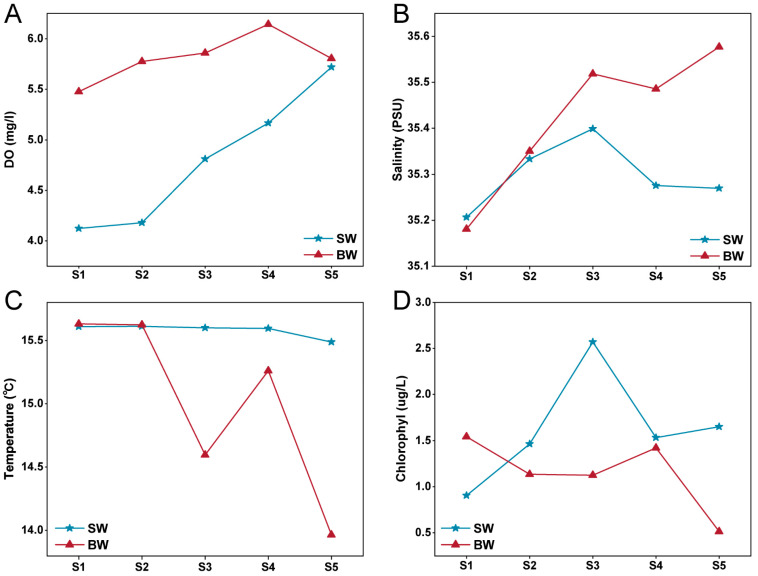
Spatial distributions of dissolved oxygen (**A**), salinity (**B**), temperature (**C**) and chlorophyll *a* concentration (**D**) in surface (SW; triangles) and bottom (BW; asterisk) waters of Ría de Vigo in sampling date. PSU, practical salinity units.

Sampling stations extended from the interior of Ría de Vigo to the open sea (Figure 1). The environmental variables measured during the study period are summarized in Table S1. From inshore to the open sea, the water temperature, salinity, DO and Chl *a* varied slightly (Table S1). To conduct a more intuitive and detailed comparative analysis among the five sites, we plotted a line chart of DO, salinity, temperature and Chl *a* (Figure 2). The water temperature of the bottom samples becomes cooler from the inshore to the open sea stations (from 15.6 to 13.9 °C). However, the DO concentrations and salinity presented the opposite pattern, increasing from 4.12 to 5.81 and from 35.20 to 35.58 from the inshore to the open sea, respectively (Figure 2). From the inshore to the open sea, the concentration of Chl *a* increased in surface waters and decreased in the bottom seawater. Spearman’s correlation analyses showed that the seawater temperature was positively correlated with longitude, latitude and Chl *a* (*p* < 0.05) and negatively correlated with depth, DO and salinity (*p* < 0.05). All detailed data are listed in Table S2.

### 3.2. The Abundances of Vibrio spp. and Total Bacteria

**Figure 3:**
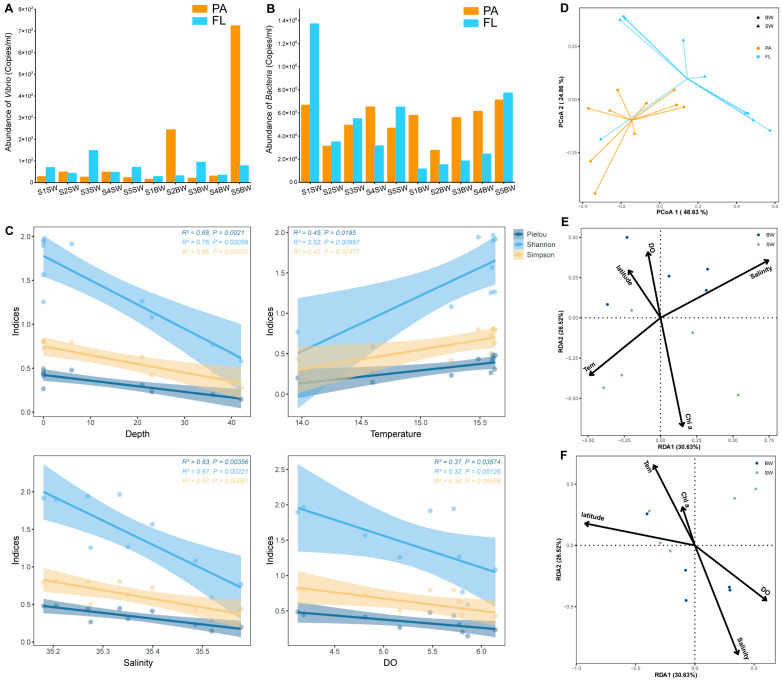
The abundance and α- and β-diversity of microbes. (**A**) The abundance of *Vibrio* spp. (**B**) The abundance of total bacteria. (**C**) The correlations of α-diversity indices with environmental factors. (**D**) The PCoA of different samples based on the Bray–Curtis dissimilarity of *Vibrio* communities. The dbRDA plot shows the relationship between FL (**E**) and PA (**F**) samples and environmental factors. Tem, temperature; DO, dissolved oxygen.

qPCR was used to estimate the abundance of the vibrionic 16S rRNA gene, which showed a slight increasing trend from the inshore to the open sea (Figure 3A and Figure S1). The highest vibrionic abundance was recorded in the FL group at S3 (1.48 × 10^2^ copies mL^−1^), and the lowest value occurred in the PA group at S5 (2.32 × 10^1^ copies mL^−1^). In the BW, samples from the outer stations tended to have higher copy numbers of the vibrionic 16S rRNA gene, and the mean value increased from 1.51 × 10^1^ (PA group at S1) to 7.24 × 10^2^ (PA group at S5) copies mL^−1^ (Figure 3A). We compared the *Vibrio* abundance on membranes with pore sizes of 3 and 0.2 μm to infer their preferential lifestyles. Overall, *Vibrio* was more abundant in the free-living (FL) than the particle-associated (PA) fraction, whereas the abundance in the particle-associated fraction was higher than that in the FL in S2BW and S5BW (Figure 3A). Total bacteria were generally more abundant in the free-living fraction in SW, whereas the particle-associated fraction was higher in the BW from S1 to S5 (Figure 3B).

### 3.3. Diversity Estimators of Vibrio spp.

The 16S rRNA partial sequences were clustered into 52 operational taxonomic units (OTUs) based on a 97% sequence similarity level. The coverages of all samples were over 99.9%, indicating that the sequences can represent the real situation of vibrios in the samples. For α-diversity indices, the evenness, richness and diversity of *Vibrio* did not show significant differences between the FL and PA groups or between SW and BW (Figure S2; *t*-test, *p* > 0.05). Correlation analyses showed significant linear relationships between the α-diversity indices and environmental factors (Figure 3C). The diversity (Shannon, Simpson) and evenness (Pielou) indexes decreased as the depth, DO and salinity increased (*p* < 0.05), and they increased as the temperature increased (*p* < 0.05). To investigate β-diversity, a principal coordinate analysis (PCoA) based on the OTU similarity matrix was performed for all samples (Figure 3D). The first two PCoA axes explained 48.63 and 24.86% of the total variation, respectively. The results demonstrate that the FL samples were clearly separated from the PA group (analysis of similarity [ANOSIM]; R = 0.3416; *p* = 0.002), indicating that the *Vibrio* community composition differed strongly between different lifestyles, whereas no significant differences were found between the SW and BW groups (Figure 3D).

### 3.4. Community Composition of Total Vibrios

**Figure 4:**
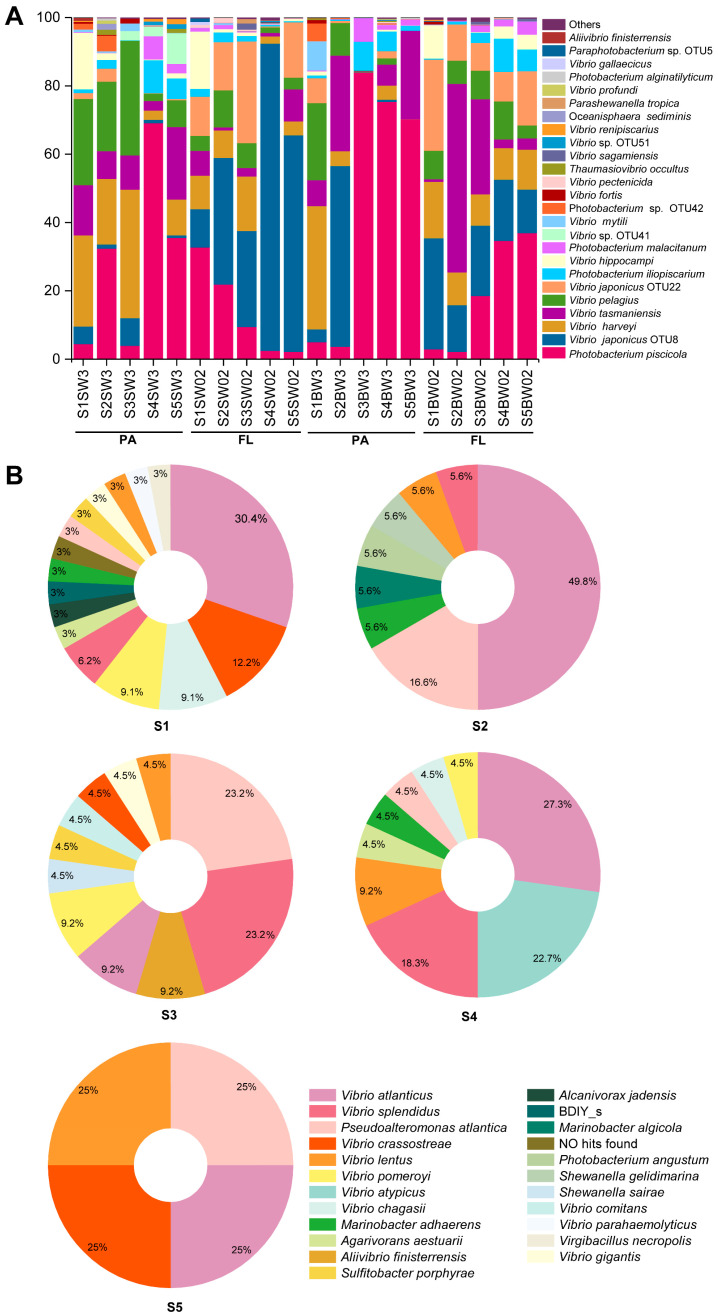
The community compositions of total and culturable *Vibrio* spp. (**A**) The top 22 abundant species across all samples. Other species comprised < 0.1% of the total samples. (**B**) The relative abundance of cultivated *Vibrio* in different sites. SW, surface seawater; BW, bottom seawater; FL, free living; PA, particle associated.

By comparing the representative sequences of each OTU against the SILVA database and EzBioCloud database, the taxonomic assignments of *Vibrio* community were conducted (Figure 4A). A total of 59.96% of the sequences belonged to *Vibrionaceae*, and 55.84% of the total sequences were assigned to the *Vibrio* genus. The abundance of the top 30 OTUs accounted for 99.74% of all species (Figure 4A). *Photobacterium piscicola* was the dominant species across all samples (27.45%) and was more abundant in the PA group than in the FL group, followed by *Vibrio japonicus* OTU8 (20.01%), *Vibrio harveyi* (11.90%) and *Vibrio tasmaniensis* (11.75%). Whether in SW or BW, the dominant species in the FL group showed similar rules, i.e., *V. japonicus* OTU8 and OTU22; *V. harveyi* and *V. tasmaniensis*; and *P. piscicola*, *V. harveyi* and *V. Pelagius* were more abundant in the PA group (Figure 4A and Figure S2). Additionally, there was a high relative abundance of *P. piscicola* in all samples and high relative abundances of *V. japonicus* OTU8, *V. harveyi* and *V. tasmaniensis* in S1BW3, S2BW3, S1BW02 and S2BW02, revealing their convertible lifestyles in the bottom seawater (Figure S3).

### 3.5. Culturable Vibrios

**Figure 5:**
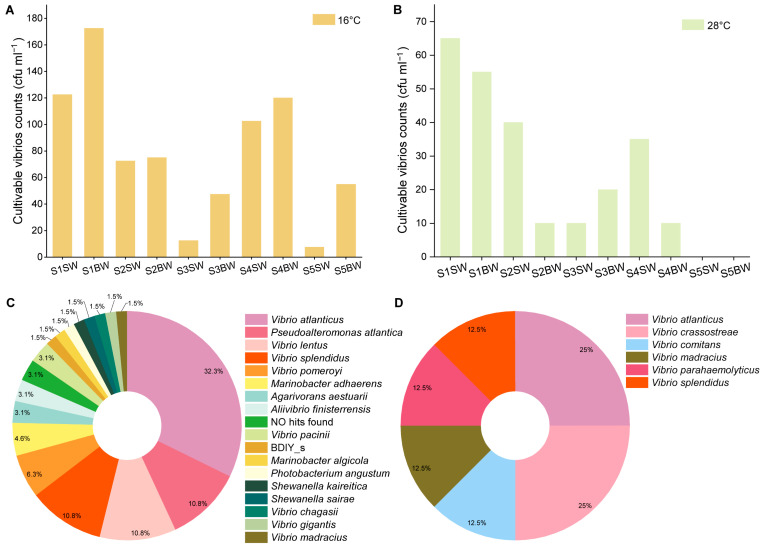
The cfu counts and species of *Vibrio* cultivated at different temperatures. This figure displays the cfu counts and distribution of species isolated at 16 °C (**A**,**C**) and 28 °C (**B**,**D**).

The spatial distribution of the colony-forming unit (cfu) counts at 16 °C and 28 °C is roughly the same (Figure 5A,B). Except for two samples (i.e., S4SW and S4BW), the numbers of cfu in most sites were higher inshore than in the open sea (Figure 5). The counts of culturable *Vibrio* spp. at 16 °C (7.5 × 10^0^–1.73 × 10^2^ cfu mL^−1^) were approximately triple compared to those at 28 °C (0–6.5 × 10^1^ cfu mL^−1^; Figure 5A,B). In total, 132 strains were isolated from Ría de Vigo. A higher number of different species were isolated under 16 °C (18) than under 28 °C (6), indicating that culture conditions closer to the in situ temperature may help to isolate more *Vibrio* species (Figure 5C,D). At 16 °C, *V. atlanticus*, *P. atlantica*, *V. lentus* and *V. splendidus* were the dominant species, whereas *V. atlanticus* and *V. crassostreae* were more abundant at 28 °C. Regarding the spatial distribution, *Vibrio atlanticus* was isolated from the five sampling stations and was the most dominant species in S1, S2, S4 and S5 (Figure 4B). *V. splendidus* was isolated from stations S1 to S4, and *P. atlantica* occupied an important position in the four sites except at S1. Interestingly, there were five bacterial strains (5.05% of the total isolates) showing low 16S rRNA gene similarities (less than 98.65) with their closet known bacterial species, representing 5 potentially novel species. Two of them were identified as novel *Vibrio* species (Figure S4), and 42 out of 52 OTUs showed similarities with these two novel vibrios (identify > 90%), showing they may belong to the same family.

### 3.6. Environmental and Stochastic Effects on Vibrio spp.

**Figure 6:**
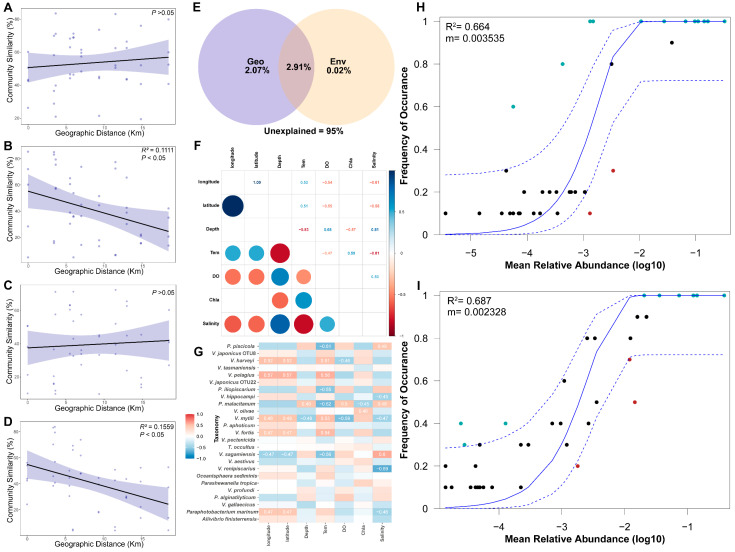
The effects of various factors on the *Vibrio* community. (**A**–**D**) The distance–decay relationships of the FL, PA, SW and BW groups. The abscissa is the geographical position, and the ordinate is the community composition. Pairwise dissimilarities (the Bray–Curtis index) are plotted as a function of the distance among sites. The data are shown as pairwise dissimilarities between the communities at 5 sites. The black line represents the best linear regression result, and the purple areas represent confidence intervals greater than 95%. (**E**) The results of the variation partitioning analysis (VPA). Env, environmental factors; Geo, spatial factors. A heat map of the Spearman’s rank correlation coefficients between environmental factors (**F**) as well as between the top 25 abundant species and factors (**G**). (**H**,**I**) A neutral model of *Vibrio* communities in the FL and PA groups, respectively.

To explore the key environmental drivers shaping the *Vibrio* community, environmental correlations were analyzed by dbRDA (Figure 3E,F). For FL or PA samples, the first and second axes explained 30.63% and 26.52% of the total variance, respectively. Monte Carlo permutation tests identified that temperature, DO, Chl *a*, salinity and latitude (*p* < 0.05; Figure 3E,F) significantly affected the *Vibrio* community structure. Salinity, temperature and Chl *a* were the main factors affecting the FL *Vibrio* populations, whereas the PA group was primarily affected by DO, salinity, latitude and temperature. To determine to what extent the environmental and spatial factors contributed to the community structure, a variation partitioning analysis (VPA) was conducted (Figure 6E). The pure effect of spatial factors (2.07%) was greater than that of environmental variables (0.02%; Figure 6E), and only 2.91% of the variation in the entire community was explained by both environmental and spatial variables (Figure 6E). It is important to note the high level of unexplained variation (95%) in the VPA. The distance–decay relationships analysis revealed that the similarities between *Vibrio* communities were affected by geographic distance (Figure 6A–D). In the PA and BW groups, lower similarity between *Vibrio* species was recorded with a larger distance (*p* < 0.05; Figure 6B,D).

A heat map of the Spearman’s rank correlation coefficients of the top 25 abundant species and environmental factors is shown in Figure 6G. The most abundant species, i.e., *P. piscicola*, was positively related to depth, DO and salinity and showed negative relations to temperature and Chl *a* (*p* < 0.05). In contrast, *V. japonicus* OTU8 was positively related to temperature and Chl *a* but negatively related to depth, DO and salinity. Indeed, DO was negatively correlated with depth and salinity, whereas Chl a showed negative relationship with temperature (Figure 6F and Table S2). Details about the correlations of the top 25 most abundant species and environmental factors are provided in Table S3. The relationship between the occurrence frequency of OTUs and their relative abundance was described by the neutral community model, indicating that the stochastic process played a crucial role in structuring *Vibrio* communities (Figure 6H,I). Stochastic processes played important roles in shaping both the FL (68.7%) and PA *Vibrio* communities (66.4%; Figure 6).

## 4. Discussion

*Vibrio* spp. are heterotrophic bacteria ubiquitously found in marine environments. Many studies on the ecological distribution of *Vibrio* have been carried out in recent years, especially in the Chinese marginal seas. Many unexplored sea areas, like the Atlantic Ocean, may harbor potential novel *Vibrio* species. Consequently, further investigations on this bacterial community in previously unexplored areas should be conducted. Based on amplicon sequencing and culture methods, our study revealed that the distribution pattern, lifestyles and environmental responses of *Vibrio* spp. were diverse in Ría de Vigo compared to other sea areas. Previous studies in the area also demonstrated that these vibrios may bloom in response to pulses of organic nutrients entering the marine environment [13], supporting their potential use as microbial indicators in eutrophication scenarios.

### 4.1. Vibrios in Ría de Vigo Have Different Strategies and May Be Microbial Indicators Under a Future Enhanced Eutrophication Scenario

As one of the important blue economy poles in Europe, with important industries in the fisheries, fish processing and shipbuilding sectors, Ría de Vigo is heavy influenced by human activities and organic matter loads [44,45]. The affinity of *Vibrio* to organic carbon substrates is quite high [16]; thus, *Vibrio* spp. may grow under high-nutrient conditions and respond rapidly to the nutrient pulses. In the present study, vibrios occurred in all seawater samples of Ría de Vigo, and the *Vibrio* abundance (2.32 × 10^1^–1.48 × 10^2^ copies mL^−1^; Figure 3A) was within the average range for estuarine and coastal waters (10^1^–10^5^ copies mL^−1^) [46]. It has been reported that *Vibrio* species, especially *V. parahaemolyticus* and *V. vulnificus*, are distributed in the seawater, mussels and shellfish of Galicia (NW Spain) [47,48,49]. This might be due to the influence of nutrients from farming activities near the sampling stations, which may have had effects on *Vibrio* ecology. Nutrient addition experiments in Ría de Vigo have shown that the combined addition of inorganic (nitrate, ammonium and phosphate) and organic nutrients (glucose and amino acids) provoked great increases in the proportion of sequence reads belonging to Vibrionales [14,50]. A similar response pattern was observed when wildfire ashes were added to seawater from Ría de Vigo, increasing the proportions of the potentially pathogenic *Vibrio* [51]. The same response, although of lower intensity, was found when DOM concentrated from natural riverine water was added to a natural microbial population in Ría de Vigo [14,15]. Due to their capacity to bloom when high DOM levels occur, *Vibrio* species may become more important as ideal microbial indicators under future enhanced eutrophication scenarios, such as the nutrition pulse from aquaculture [52].

*Vibrio* populations exhibit two alternative growth strategies, i.e., free-living bacterioplankton and associations with marine particles and/or living hosts [16]. Previous studies have reported that vibrios may act as mutual symbionts or disease agents so they may adopt a particle-associated lifestyle as their main growth strategy to take up sufficient nutrients [16,53]. However, in all SW and almost all BW samples from Ría de Vigo, *Vibrio* was more abundant in the FL fraction than in the PA fraction (Figure 2A). The preference for an FL lifestyle at a particular time or in a particular sea area has also been found in other studies, such as the Northern Chinese marginal seas, the Sansha Yongle Blue Hole, the ECS and SCS, and the Baltic and Skagerrak Seas [16,54,55,56]. And, in the ETIO, vibrios show a shift from the FL lifestyle to mixed lifestyles from the surface to bottom seawater. These findings may be related to the lower seawater temperature measured during sampling in Ría de Vigo (<16 °C; Figure 2C), as the FL state may help vibrios find appropriate trophic niches through chemotactic motility [57]. Indeed, temperature is likely the most important driver of the overall change in *Vibrio* abundance in temperate coastal waters, and increased sea surface temperature may support the view that ocean warming favors the spread of vibrios [58,59]. However, mechanisms determining when *Vibrio* remains in the FL or PA lifestyle are still unknown and should be further investigated.

Through traditional plating techniques, microbiologists have obtained pure cultures since the eighteenth century [60]. The counts of culturable *Vibrio* spp. at 16 °C were approximately three times higher than those at 28 °C (Figure 5A,B), and more species were isolated from 16 °C (18) than from 28 °C (6), indicating that culture conditions closer to in situ temperatures result in the isolation of more *Vibrio* species. Indeed, since the natural milieu usually contains every nutrient required for bacterial growth, greater attention should be paid to mimicking in situ conditions in future microbial isolation studies [61]. Additionally, the CFUs at sites S1–S3 showed slightly higher counts than gene copies without a change in the order of magnitude, which might have been due to the limited selectivity of TCBS agar, which can support the growth of other bacteria (e.g., *Shewanella* and *Halomonas*) [34].

### 4.2. A Diverse Community of Vibrio Species Has Been Recorded in Ría de Vigo

Dominant *Vibrio* populations may differ among various marine regions due to variable environmental conditions. In seawater, it has been reported that the most prominent species in YS are *V. chagasii* and *V. harveyi*, whereas *V. japonicus* and *V. chagasii* are the dominant groups in the ECS and SCS, respectively [16], and *V. rotiferianus* is the most abundant species in the eastern tropical Indian Ocean [22]. In this study, *P. piscicola* was the dominant species across all samples, followed by *V. japonicus*, *V. harveyi* and *V. tasmaniensis* (Figure 4A). Our results show that the relative abundances of *P. piscicola* and *V. tasmaniensis* were significantly higher in Ría de Vigo than in other temperate seas, likely due to the special set of environmental conditions which were selected for specific species to adapt and bloom [13,62]. Although no differences were observed in terms of overall fish health status, the strain of *P. piscicola* was first isolated from whiting from the North Sea (Netherlands), and the fish digesta property was highly associated with the dominance of *P. piscicola* [63,64]. *P. piscicola* had wide physiological characteristics like the optimal temperature for growth and the substrate utilization spectrum [63], contributing to its high adaptation in Ría de Vigo. *V. tasmaniensis* was first isolated from Atlantic salmon (*Salmo salar* L.) and can become a model pathogen of oysters, and it is also pathogenic to blue mussel larva, which may be associated with nutrient addition from farming activities [65,66]. Additionally, it can grow in the temperature range of 4–35 °C [65], a thermal range that includes the temperature range in Ría de Vigo during sampling (13.9–15.6 °C; Table S1). In addition, stochasticity played a significant role in driving the assembly of the marine *Vibrio* community along Ría de Vigo (Figure 6H,I), meaning that some unmeasured factors may also influence *Vibrio* communities [67].

*Vibrio* spp. may evolve several adaptive strategies to survive in various environments based on their genetically and metabolically diverse nature [16]. The most common species across all samples, i.e., *V. japonicus* and *V. harveyi*, comprised the highest proportions in Ría de Vigo and other sea areas, indicating that they may adapt to a wide range of environments. It has been reported that *V. harveyi* is a serious pathogen of marine vertebrates and invertebrates, leading to eye lesions/blindness, gastro-enteritis, muscle necrosis, skin ulcers and tail rot disease [5,8,9]. Highly productive shellfish farming activities are carried out in Ría de Vigo, with more than 37,000 tons of mussels produced annually [28]. Thus, the high relative abundance of *V. harveyi* may increase the risk of pathogenic infection in the local aquaculture ecosystem. And many *V. harveyi* strains can utilize various mono- and polysaccharides (e.g., fructose, mannose and dextrose) and tolerate a wide range of salinities, and they have the ability to attach and form biofilms (quorum sensing, lipopolysaccharide and extracellular products) [8,9], suggesting that they may have a strong competitive advantage in global oceans. Additionally, the relative abundance of *V. japonicus* was significantly higher in Ría de Vigo, likely due to its optimal temperature (10–37 °C) [68]. Future studies should simulate pathogenic experiments on vibrios within aquaculture ecosystems.

More than 99% of microbial species remain undiscovered to date, and only a small fraction can be cultured using current techniques [69,70]. To isolate more unculturable bacteria from complex environments, several methods have been developed in recent years, including the use of dilute nutrient media specifically designed for the growth of bacteria adapted to oligotrophic conditions and the provision of simulated natural environmental conditions for bacterial culture [61]. In this study, five potentially novel bacteria were detected among isolates from Ría de Vigo, representing a higher proportion (3.8% of the total isolates) compared to other reported areas, such as coral reef regions in the SCS (2.4%) [34]. Our study may also provide implications for the discovery of novel species, i.e., isolating strains in previously unexplored sea areas worldwide.

In this study, the fact that many sequences did not belong to the genus *Vibrio* after annotation was likely due to the limitation of primers, which often lack highly variable domains to separate closely related organisms [71]. After re-annotation with the EzBioCloud database, only 19 out of 52 OTUs could be accurately annotated. 16S rRNA gene analysis may have some limitations because it does not contain highly variable domains for the closely related organisms [71]. Protein-coding marker genes, such as heat shock protein 60 (*hsp60*) [72], ferric uptake regulator (*fur*) [73] and uridylate kinase (*pyrH*) [74], have been proposed as more reliable markers for differentiating *Vibrio* species. In our laboratory experiments, we have made many attempts to amplify these genes, but it is difficult to successfully amplify environmental DNA. Identifying more accurate marker genes for sequencing will significantly improve the study of *Vibrio* spp. in the future.

## 5. Conclusions

The ecological distribution of total and culturable *Vibrio* spp. in seawater along Ría de Vigo was investigated by cultivable methods, qPCR and high-throughput sequencing. A slight increase in the abundance of *Vibrio* spp. was recorded from the inshore to the open sea, with a preference for a temporary free-living lifestyle. The community compositions had differences between the FL and PA groups and were mainly affected by the stochastic process. *V. harveyi*, a pathogen of maricultural fish and invertebrates, emerged as the dominant species across all samples in Ría de Vigo. Our study provides valuable insights into the management and pathogen prevention of aquatic aquaculture, and in the future, more samples should be collected from the global ocean to explore the role of *Vibrio* spp. in the aquaculture ecosystem.

## Data Availability

The GenBank accession numbers for the 16S rRNA gene sequence of the three potential novel vibrios are PQ357159 and PQ357160, respectively. And the raw data files for *Vibrio* spp. are attached in the Supplementary Materials, including the OTU table and representative sequences.

## Supplementary Materials

The following supporting information can be downloaded at https://www.mdpi.com/article/10.3390/biology13120986/s1, Figure S1: The standard curves of total bacteria (A) and *Vibrio* spp. (B) for qPCR quantification. The blue points represent each sample; Figure S2: The α-diversity indices of the *Vibrio* community. The differences in values between FL and PA (A), BW and SW in FL (B) and PA (C) samples were calculated using Student’s *t*-test; Figure S3: The heat map of the relative abundance of *Vibrio* spp. in each sample. FL: free-living group, PA: particle-associated group; Figure S4: A neighbor-joining phylogenetic tree based on 16S rRNA gene sequences showing the phylogenetic position of potentially novel strains and other closely related species. Percentage bootstrap values above 50% (1000 replicates) are shown at branch nodes. Bar, 0.01 nucleotide substitutions per nucleotide position; Table S1: The environmental parameters in 5 sampling sites; Table S2: Spearman’s rank correlation coefficients among and between environmental factors and microbial abundance; Table S3: Spearman’s rank correlation coefficients between top 25 abundant species and environmental factors; Table S4: Spearman’s rank correlation coefficients between alpha diversity indices and environmental factors.

## References

[1] Siboni N., Balaraju V., Carney R., Labbate M., Seymour J.R. (2016). Spatiotemporal dynamics of *Vibrio* spp. within the Sydney Harbour estuary. Front. Microbiol..

[2] Thompson J.R., Randa M.A., Marcelino L.A., Tomita-Mitchell A., Lim E., Polz M.F. (2004). Diversity and dynamics of a North Atlantic coastal Vibrio community. Appl. Environ. Microbiol..

[3] Liang J., Liu J., Wang X., Lin H., Liu J., Zhou S., Sun H., Zhang X.-H. (2019). Spatiotemporal dynamics of free-living and particle-associated Vibrio communities in the northern Chinese marginal seas. Appl. Environ. Microbiol..

[4] Baker-Austin C., Oliver J.D., Alam M., Ali A., Waldor M.K., Qadri F., Martinez-Urtaza J. (2018). *Vibrio* spp. infections. Nat. Rev. Dis. Primers.

[5] Zhang X.-H., He X., Austin B. (2020). Vibrio harveyi: A serious pathogen of fish and invertebrates in mariculture. Mar. Life Sci. Technol..

[6] Wang G., Shuai L., Li Y., Lin W., Zhao X., Duan D. (2008). Phylogenetic Analysis of Epiphytic Marine Bacteria on Hole-Rotten Diseased Sporophytes of Laminaria Japonica. J. Appl. Phycol..

[7] Reidl J., Klose K.E. (2002). Vibrio cholerae and cholera: Out of the water and into the host. FEMS Microbiol. Rev..

[8] Montánchez I., Kaberdin V.R. (2020). Vibrio harveyi: A brief survey of general characteristics and recent epidemiological traits associated with climate change. Mar. Environ. Res..

[9] Austin B., Zhang X.-H. (2006). Vibrio harveyi: A significant pathogen of marine vertebrates and invertebrates. Lett. Appl. Microbiol..

[10] Jawdhari A., Deák G., Mihăilescu D.F., Crăciun N., Staicu A.C., Stanca I., Cozorici D., Fendrihan S., Pop C.-E., Mernea M. (2024). Ingested Microplastics Can Act as Microbial Vectors of Ichthyofauna. Microbiol. Res..

[11] Zhu J., Xu M., Liu Q., Li D., Yang R., Chen H. (2021). Bacteriophage therapy on the conchocelis of *Pyropia haitanensis* (Rhodophyta) infected by Vibrio mediterranei 117-T6. Aquaculture.

[12] Wang X., Liu J., Liang J., Sun H., Zhang X.-H. (2020). Spatiotemporal dynamics of the total and active Vibrio spp. populations throughout the Changjiang estuary in China. Environ. Microbiol..

[13] Zhang X.-H., Lin H., Wang X., Austin B. (2018). Significance of Vibrio species in the marine organic carbon cycle—A review. Sci. China Earth Sci..

[14] Gutiérrez-Barral A., Teira E., Hernández-Ruiz M., Fernández E. (2021). Response of prokaryote community composition to riverine and atmospheric nutrients in a coastal embayment: Role of organic matter on Vibrionales. Estuar. Coast. Shelf Sci..

[15] Baffone W., Tarsi R., Pane L., Campana R., Repetto B., Mariottini G.L., Pruzzo C. (2006). Detection of free-living and plankton-bound vibrios in coastal waters of the Adriatic Sea (Italy) and study of their pathogenicity-associated properties. Environ. Microbiol..

[16] Wang X., Liu J., Zhao W., Liu J., Liang J., Thompson F., Zhang X.-H. (2022). Fine-scale structuring of planktonic *Vibrio* spp. in the Chinese marginal seas. Appl. Environ. Microbiol..

[17] Pedrós-Alió C. (2012). The rare bacterial biosphere. Annu. Rev. Mar. Sci..

[18] Walters K.E., Martiny J.B. (2020). Alpha-, beta-, and gamma-diversity of bacteria varies across habitats. PLoS ONE.

[19] Liang J., Liu J., Wang X., Sun H., Zhang Y., Ju F., Thompson F., Zhang X.-H. (2022). Genomic analysis reveals adaptation of Vibrio campbellii to the hadal ocean. Appl. Environ. Microbiol..

[20] Hasan N.A., Grim C.J., Lipp E.K., Rivera I.N., Chun J., Haley B.J., Taviani E., Choi S.Y., Hoq M., Munk A.C. (2015). Deep-sea hydrothermal vent bacteria related to human pathogenic Vibrio species. Proc. Natl. Acad. Sci. USA.

[21] Smriga S., Fernandez V.I., Mitchell J.G., Stocker R. (2016). Chemotaxis toward phytoplankton drives organic matter partitioning among marine bacteria. Proc. Natl. Acad. Sci. USA.

[22] Zhu S., Wang X., Zhao W., Zhang Y., Song D., Cheng H., Zhang X.-H. (2023). Vertical dynamics of free-living and particle-associated vibrio communities in the eastern tropical Indian Ocean. Front. Microbiol..

[23] Liang J., Liu J., Zhan Y., Zhou S., Xue C.-X., Sun C., Lin Y., Luo C., Wang X., Zhang X.-H. (2021). Succession of marine bacteria in response to Ulva prolifera-derived dissolved organic matter. Environ. Int..

[24] Martínez A., Ventouras L.A., Wilson S.T., Karl D.M., DeLong E.F. (2013). Metatranscriptomic and functional metagenomic analysis of methylphosphonate utilization by marine bacteria. Front. Microbiol..

[25] Li N., Dong K., Jiang G., Tang J., Xu Q., Li X., Kang Z., Zou S., Chen X., Adams J.M. (2020). Stochastic processes dominate marine free-living Vibrio community assembly in a subtropical gulf. FEMS Microbiol. Ecol..

[26] Fernández E., Álvarez-Salgado X.A., Beiras R., Ovejero A., Méndez G. (2016). Coexistence of urban uses and shellfish production in an upwelling-driven, highly productive marine environment: The case of the Ría de Vigo (Galicia, Spain). Reg. Stud. Mar. Sci..

[27] Diz P., Francés G., Vilas F. (2000). Microhábitats de foraminíferos bentónicos en la ría de Vigo y su aplicación a la interpretación paleoecológica. J. Iber. Geol..

[28] Surís-Regueiro J.C., Garza-Gil M.D., Varela-Lafuente M.M. (2014). Socio-economic quantification of fishing in a European urban area: The case of Vigo. Mar. Policy.

[29] Perez-Arlucea M., Mendez G., Clemente F., Nombela M., Rubio B., Filgueira M. (2005). Hydrology, sediment yield, erosion and sedimentation rates in the estuarine environment of the Ria de Vigo, Galicia, Spain. J. Mar. Syst..

[30] Evans G., Prego R., Marshall J.E. (2011). Organic matter in ria sediments: Relevance of terrestial sources and temporal variations in rates of accumulation. Estuar. Coast. Shelf Sci..

[31] Teira E., Hernández-Ruiz M., Barber-Lluch E., Sobrino C., Teixeira I., Álvarez-Salgado X.A., Nieto-Cid M., Martínez-García S., Figueiras F., Fernández E. (2016). Bacterioplankton responses to riverine and atmospheric inputs in a coastal upwelling system (Ría de Vigo, NW Spain). Mar. Ecol. Prog. Ser..

[32] Zhang Y., Zhao Z., Dai M., Jiao N., Herndl G.J. (2014). Drivers shaping the diversity and biogeography of total and active bacterial communities in the South China Sea. Mol. Ecol..

[33] Ruiz Y., Suarez P., Alonso A., Longo E., Villaverde A., San Juan F. (2011). Environmental quality of mussel farms in the Vigo estuary: Pollution by PAHs, origin and effects on reproduction. Environ. Pollut..

[34] Liu M., Yin F., Zhao W., Tian P., Zhou Y., Jia Z., Huang K., Ding Y., Xiao J., Niu W. (2024). Diversity of Culturable Bacteria from the Coral Reef Areas in the South China Sea and Their Agar-Degrading Abilities. Microorganisms.

[35] Zhang Z., Yu T., Xu T., Zhang X.-H. (2014). Aquimarina pacifica sp. nov., isolated from seawater. Int. J. Syst. Evol. Microbiol..

[36] Thompson J.D., Gibson T.J., Plewniak F., Jeanmougin F., Higgins D.G. (1997). The CLUSTAL_X windows interface: Flexible strategies for multiple sequence alignment aided by quality analysis tools. Nucleic Acids Res..

[37] Zhao W., Chen X., Liu R., Tian P., Niu W., Zhang X.-H., Liu J., Wang X. (2023). Distinct coral environments shape the dynamic of planktonic Vibrio spp. Environ. Microbiome.

[38] Chen S., Zhou Y., Chen Y., Gu J. (2018). fastp: An ultra-fast all-in-one FASTQ preprocessor. Bioinformatics.

[39] Magoč T., Salzberg S.L. (2011). FLASH: Fast length adjustment of short reads to improve genome assemblies. Bioinformatics.

[40] Callahan B.J., McMurdie P.J., Rosen M.J., Han A.W., Johnson A.J.A., Holmes S.P. (2016). DADA2: High-resolution sample inference from Illumina amplicon data. Nat. Methods.

[41] Hall M., Beiko R.G. (2018). 16S rRNA gene analysis with QIIME2. Microbiome Anal. Methods Protoc..

[42] Burns A.R., Stephens W.Z., Stagaman K., Wong S., Rawls J.F., Guillemin K., Bohannan B.J. (2016). Contribution of neutral processes to the assembly of gut microbial communities in the zebrafish over host development. ISME J..

[43] Mo Y., Peng F., Gao X., Xiao P., Logares R., Jeppesen E., Ren K., Xue Y., Yang J. (2021). Low shifts in salinity determined assembly processes and network stability of microeukaryotic plankton communities in a subtropical urban reservoir. Microbiome.

[44] Barton E.D., Largier J., Torres R., Sheridan M., Trasviña A., Souza A., Pazos Y., Valle-Levinson A. (2015). Coastal upwelling and downwelling forcing of circulation in a semi-enclosed bay: Ria de Vigo. Prog. Oceanogr..

[45] Vilas F., Nombela M., García-Gil E., García-Gil S., Alejo I., Rubio B., Pazos O. (1995). Mapa de distribución de los sedimentos del fondo de la Ría de Vigo. Cons. Pesca Marisqueo Acuic. Xunta Galicia.

[46] Thompson J.R., Polz M.F., Thompson F.L., Austin B., Swings. J. (2006). Dynamics of *Vibrio* populations and their role in environmental nutrient cycling. The Biology of Vibrios.

[47] Romero A., del Mar Costa M., Forn-Cuni G., Balseiro P., Chamorro R., Dios S., Figueras A., Novoa B. (2014). Occurrence, seasonality and infectivity of Vibrio strains in natural populations of mussels Mytilus galloprovincialis. Dis. Aquat. Org..

[48] Martinez-Urtaza J., Blanco-Abad V., Rodriguez-Castro A., Ansede-Bermejo J., Miranda A., Rodriguez-Alvarez M.X. (2012). Ecological determinants of the occurrence and dynamics of Vibrio parahaemolyticus in offshore areas. ISME J..

[49] Garrido-Maestu A., Lozano-León A., Rodríguez-Souto R.R., Vieites-Maneiro R., Chapela M.J., Cabado A.G. (2016). Presence of pathogenic Vibrio species in fresh mussels harvested in the southern Rias of Galicia (NW Spain). Food Control.

[50] Gutiérrez-Barral A., Fernández E., Hernández-Ruiz M., Teira E. (2024). Contrasting resistance of prokaryotic plankton biomass and community composition to experimental nutrient inputs in a coastal upwelling system (NW Spain). Hydrobiologia.

[51] Gutiérrez-Barral A., Teira E., Díaz-Alonso A., Justel-Díez M., Kaal J., Fernández E. (2024). Impact of wildfire ash on bacterioplankton abundance and community composition in a coastal embayment (Ría de Vigo, NW Spain). Mar. Environ. Res..

[52] Xu W., Gong L., Yang S., Gao Y., Ma X., Xu L., Chen H., Luo Z. (2020). Spatiotemporal dynamics of Vibrio communities and abundance in Dongshan Bay, South of China. Front. Microbiol..

[53] Lin H., Yu M., Wang X., Zhang X.-H. (2018). Comparative genomic analysis reveals the evolution and environmental adaptation strategies of vibrios. Bmc Genom..

[54] Li B., Liu J., Zhou S., Fu L., Yao P., Chen L., Yang Z., Wang X., Zhang X.-H. (2020). Vertical variation in Vibrio community composition in Sansha Yongle Blue Hole and its ability to degrade macromolecules. Mar. Life Sci. Technol..

[55] Eiler A., Johansson M., Bertilsson S. (2006). Environmental influences on Vibrio populations in northern temperate and boreal coastal waters (Baltic and Skagerrak Seas). Appl. Environ. Microbiol..

[56] Unanue M., Ayo B., Azúa I., Barcina I., Iriberri J. (1992). Temporal variability of attached and free-living bacteria in coastal waters. Microb. Ecol..

[57] Brennan C.A., DeLoney-Marino C.R., Mandel M.J. (2013). Chemoreceptor VfcA mediates amino acid chemotaxis in Vibrio fischeri. Appl. Environ. Microbiol..

[58] Vezzulli L., Brettar I., Pezzati E., Reid P.C., Colwell R.R., Höfle M.G., Pruzzo C. (2012). Long-term effects of ocean warming on the prokaryotic community: Evidence from the vibrios. ISME J..

[59] Asplund M.E., Rehnstam-Holm A.S., Atnur V., Raghunath P., Saravanan V., Härnström K., Collin B., Karunasagar I., Godhe A. (2011). Water column dynamics of Vibrio in relation to phytoplankton community composition and environmental conditions in a tropical coastal area. Environ. Microbiol..

[60] Bodor A., Bounedjoum N., Vincze G.E., Erdeiné Kis Á., Laczi K., Bende G., Szilágyi Á., Kovács T., Perei K., Rákhely G. (2020). Challenges of unculturable bacteria: Environmental perspectives. Rev. Environ. Sci. Bio/Technol..

[61] Vartoukian S.R., Palmer R.M., Wade W.G. (2010). Strategies for culture of ‘unculturable’ bacteria. FEMS Microbiol. Lett..

[62] Wang X., Liu J., Li B., Liang J., Sun H., Zhou S., Zhang X.-H. (2019). Spatial heterogeneity of Vibrio spp. in sediments of Chinese marginal seas. Appl. Environ. Microbiol..

[63] Figge M.J., Cleenwerck I., van Uijen A., De Vos P., Huys G., Robertson L. (2014). Photobacterium piscicola sp. nov., isolated from marine fish and spoiled packed cod. Syst. Appl. Microbiol..

[64] Zhao R., Symonds J.E., Walker S.P., Steiner K., Carter C.G., Bowman J.P., Nowak B.F. (2021). Effects of feed ration and temperature on *Chinook salmon* (*Oncorhynchus tshawytscha*) microbiota in freshwater recirculating aquaculture systems. Aquaculture.

[65] Thompson F.L., Thompson C., Swings J. (2003). *Vibrio tasmaniensis* sp. nov., isolated from Atlantic salmon (*Salmo salar* L.). Syst. Appl. Microbiol..

[66] Islam S.S., Zhang S., Eggermont M., Bruto M., Le Roux F., Defoirdt T. (2022). The impact of the multichannel quorum sensing systems of *Vibrio tasmaniensis* and *Vibrio crassostreae* on virulence towards blue mussel (*Mytilus edulis*) larvae. Aquaculture.

[67] Malard L.A., Anwar M.Z., Jacobsen C.S., Pearce D.A. (2019). Biogeographical patterns in soil bacterial communities across the Arctic region. FEMS Microbiol. Ecol..

[68] Ishimaru K., Akagawa-Matsushita M., Muroga K. (1995). *Vibrio penaeicida* sp. nov., a pathogen of kuruma prawns (*Penaeus japonicus*). Int. J. Syst. Evol. Microbiol..

[69] Hahn M.W., Koll U., Schmidt J. (2019). Isolation and cultivation of bacteria. Struct. Funct. Aquat. Microb. Communities.

[70] Locey K.J., Fisk M.C., Lennon J.T. (2017). Microscale insight into microbial seed banks. Front. Microbiol..

[71] Ruimy R., Breittmayer V., Elbaze P., Lafay B., Boussemart O., Gauthier M., Christen R. (1994). Phylogenetic analysis and assessment of the genera *Vibrio*, *Photobacterium*, *Aeromonas*, and *Plesiomonas* deduced from small-subunit rRNA sequences. Int. J. Syst. Evol. Microbiol..

[72] Jesser K., Noble R. (2018). Characterizing the ecology of Vibrio in the Neuse River Estuary, North Carolina using heat shock protein 60 (hsp60) next-generation amplicon sequencing. Appl Env. Microbiol.

[73] Machado H., Gram L. (2015). The fur gene as a new phylogenetic marker for Vibrionaceae species identification. Appl. Environ. Microbiol..

[74] Amin A.R., Feng G., Al-Saari N., Meirelles P.M., Yamazaki Y., Mino S., Thompson F.L., Sawabe T., Sawabe T. (2016). The first temporal and spatial assessment of Vibrio diversity of the surrounding seawater of coral reefs in Ishigaki, Japan. Front. Microbiol..

