# Supplementary material for: A Diverse Vibrio Community in Ría de Vigo (Northwestern Spain)
Source: Biology (Basel). 2024 Nov 28;13(12):986. doi: 10.3390/biology13120986 (PMC11726738; doi:10.3390/biology13120986)
Supplement: Supplementary file 1 [file biology-13-00986-s001.zip › biology-3322259-supplementary.pdf]

Supplementary materials for:

A diverse *Vibrio* community in the Ría de Vigo (Northwestern Spain)

Xiaoyun Huang[1,3†], Keyi Huang[1,3†], Sihan Chen[1,3], Xinglan Yin[1,3], María Pérez-Lorenzo[2], Eva Teira[2], Emilio Fernández[2*] and Xiaolei Wang[1,3*]

1 College of Marine Life Sciences, Frontiers Science Center for Deep Ocean Multispheres and Earth System, Ocean University of China, Qingdao 266003, China

2 Centro de Investigación Mariña da Universidade de Vigo, Departamento de Ecoloxía e Bioloxía Animal, Facultade de Ciencias do Mar, Universidade de Vigo, Vigo, Galicia, Spain

3 Institute of Evolution & Marine Biodiversity, Ocean University of China, Qingdao 266100, China

***Author for correspondence:**

Xiaolei Wang

Email: wangxiaolei@ouc.edu.cn

Emilio Fernández

Email: esuarez@uvigo.gal

[†]These authors have contributed equally to this work.

Running title: A diverse *Vibrio* community.

Supplementary tables:

Table S1. The environmental parameters in 5 sampling sites.

**Samples**	**Longitude (°W)**	**Latitude (°N)**	**Depth (m)**	**Tem (°C)**	**Salinity (PSU)**	**DO (mg/l)**	**Chl *a* (μg/l)**
**S1SW**	8.6426	42.2946	1	15.6	35.20	4.12	0.90
**S1BW**	8.6426	42.2946	6	15.6	35.18	5.48	1.54
**S2SW**	8.7010	42.2700	1	15.6	35.33	4.18	1.46
**S2BW**	8.7010	42.2700	21	15.6	35.35	5.77	1.13
**S3SW**	8.7615	42.2404	1	15.6	35.40	4.81	2.57
**S3BW**	8.7615	42.2404	42	14.6	35.52	5.86	1.12
**S4SW**	8.7911	42.2189	1	15.5	35.28	5.17	1.53
**S4BW**	8.7911	42.2189	23	15.2	35.49	6.14	1.42
**S5SW**	8.8285	42.2016	1	15.4	35.27	5.72	1.65
**S5BW**	8.8285	42.2016	36	13.9	35.58	5.81	0.51

*Tem, temperature.

Table S2. Spearman's rank correlation coefficients among environmental and between environmental factors and microbial abundance.

Environmental factors	Longitude (°W)	Latitude (°N)	Depth (m)	Tem (℃)	DO (mg/l)	Chl *a* (μg/l)	Salinity (PSU)	*Vibrio* spp.		Bacteria	
								PA	FL	PA	FL
**Longitude**	1.00	**1.00**	-0.31	**0.52**	**-0.54**	-0.04	**-0.61**	-0.30	-0.44	-0.20	-0.32
**Latitude(°N)**	**1.00**	1.00	-0.29	**0.51**	**-0.55**	-0.02	**-0.58**	-0.30	-0.44	-0.20	-0.32
**Depth (m)**	-0.31	-0.29	1.00	**-0.83**	**0.68**	**-0.57**	**0.81**	0.07	-0.006	0.19	-0.41
**Tem (℃)**	**0.52**	**0.51**	**-0.83**	1.00	**-0.47**	**0.59**	**-0.81**	-0.15	**-0.64**	-0.45	-0.37
**DO (mg/l)**	**-0.54**	**-0.55**	**0.68**	**-0.47**	1.00	-0.19	**0.50**	0.05	-0.03	0.05	-0.41
**Chl *a* (μg/l)**	-0.04	-0.02	**-0.57**	**0.59**	-0.19	1.00	-0.26	-0.45	-0.03	-0.45	-0.19
**Salinity (PSU)**	**-0.61**	**-0.58**	**0.81**	**-0.81**	**0.50**	-0.26	1.00	0.39	0.47	0.09	0.03

*-, negative correlation; Bold, $P < 0.05$; Tem, temperature; PA, particle-associated; FL, free-living.

Table S3. Spearman's rank correlation coefficients between top 25 abundant species and environmental factors.

Taxonomy	Longitude (E°)	Latitude (N°)	Depth (m)	T (°C)	DO (mg/l)	Chl *a* (μg/l)	Salinity (PSU)
Aliivibrio finisterrensis	0.228035085	0.228035085	-0.119769894	0.166255904	-0.142505061	-0.017273341	-0.347625981
Paraphotobacterium	**0.470751153**	**0.470751153**	-0.132897208	0.338980949	-0.301316399	-0.0753291	**-0.460666418**
Vibrio gallaecicus	-0.056407607	-0.056407607	-0.359753112	-0.003967598	-0.234088282	0.047611176	-0.273764262
Photobacterium	-0.302292683	-0.302292683	0.238157844	-0.142223578	0.408479347	-0.142223578	0.267909531
Vibrio profundi	0.009862873	0.009862873	0.196848919	-0.140828147	0.184533434	-0.106835146	0.247663293
Parashewanella tropica	0	0	-0.213007161	0.039936153	-0.199680766	0.359425379	0.11980846
Oceanisphaera sediminis	0.235375577	0.235375577	-0.074175186	0.344775495	-0.130377288	-0.052150915	-0.002897273
Vibrio renipiscarius	0.0625	0.0625	-0.343038056	0.250798913	-0.201562316	0.129246066	**-0.69392828**
Vibrio aestivus	-0.298591934	-0.298591934	-0.097273167	-0.154460379	0.1414336	0.277284295	-0.195401684
Vibrio sagamiensis	**-0.467880238**	**-0.467880238**	0.298671946	**-0.559972186**	0.317199434	-0.02303683	**0.598957591**
Thaumasiovibrio occultus	0.00805823	0.00805823	0.025394337	0.031740784	0.107125146	0.17854191	-0.111092744
Vibrio pectenicida	-0.028506145	-0.028506145	0.064879364	-0.226126787	0.032749397	-0.115402636	0.191817895
Vibrio fortis	**0.467217039**	**0.467217039**	-0.237322226	**0.537268602**	-0.296632806	0.357170113	-0.370791007
Photobacterium aphoticum	0.266193939	0.266193939	-0.030229601	0.269214257	-0.120437957	-0.134607128	-0.191283814
Vibrio mytili	**0.490880694**	**0.490880694**	**-0.488252631**	**0.5302139**	**-0.586105394**	0.406346265	**-0.466769502**
Vibrio olivae	-0.251805078	-0.251805078	-0.290959913	0.042507514	-0.017711464	**0.456955778**	-0.191283814
Photobacterium malacitanum	-0.416934751	-0.416934751	**0.486275203**	**-0.62189122**	**0.495097865**	**-0.446795634**	**0.483022307**
Vibrio hippocampi	0.248414839	0.248414839	-0.257726154	0.134391112	-0.341262823	-0.090600749	**-0.451493735**
Photobacterium iliopiscarium	-0.349489424	-0.349489424	0.328477289	**-0.549437874**	0.371323399	-0.350191173	0.428682297

Vibrio japonicus OTU22	0.232992949	0.232992949	-0.112712795	0.283775605	-0.132831134	0.150944471	-0.217360038
Vibrio pelagius	**0.570219586**	**0.570219586**	-0.302714365	**0.576607879**	-0.404531182	0.271700048	-0.341134504
Vibrio tasmaniensis	-0.024525574	-0.024525574	0.090170236	0.042264452	0.10264224	-0.211322259	0.066415567
Vibrio harveyi	**0.515037045**	**0.515037045**	-0.325256924	**0.510192312**	**-0.464908971**	0.289813384	-0.371323399
Vibrio japonicus OTU8	0.147153441	0.147153441	-0.231866322	0.326040057	-0.169057807	0.256605601	-0.217360038
Photobacterium piscicola	-0.36175221	-0.36175221	0.380003139	**-0.606796773**	0.341134504	-0.392455624	**0.486041196**

*-, negative correlation; Bold, $P < 0.05$.

Table S4. Spearman's rank correlation coefficients between alpha diversity indices and environmental factors.

Taxonomy	Environment	Particle-associated group		Free-living group	
		r	r_sig	r	r_sig
Pielou	Depth	-0.730554248	-0.73	0.368509665	NA
	Tem	0.76969697	0.77	-0.333333333	NA
	DO	-0.793939394	-0.79	0.078787879	NA
	Chl *a*	0.345454545	NA	-0.672727273	-0.67
	Salinity	-0.866666667	-0.87	0.43030303	NA
Sobs	Depth	-0.188634096	NA	-0.175624848	NA
	Tem	0.024390697	NA	0.310981391	NA
	DO	0.170734881	NA	-0.152441858	NA
	Chl *a*	0.274395345	NA	0.396348832	NA
	Salinity	-0.231711625	NA	-0.237809299	NA
Ace	Depth	-0.006465082	NA	-0.226277864	NA
	Tem	-0.212121212	NA	0.393939394	NA
	DO	0.333333333	NA	-0.127272727	NA
	Chl *a*	0.163636364	NA	0.466666667	NA
	Salinity	-0.03030303	NA	-0.296969697	NA
Chao	Depth	-0.122836555	NA	-0.148696882	NA
	Tem	-0.054545455	NA	0.345454545	NA
	DO	0.224242424	NA	-0.115151515	NA

	Chl *a*	0.187878788	NA	0.321212121	NA
	Salinity	-0.163636364	NA	-0.248484848	NA
**Simpson**	Depth	-0.795205066	-0.8	0.536601793	NA
	Tem	0.648484848	0.65	-0.36969697	NA
	DO	-0.842424242	-0.84	0.248484848	NA
	Chl *a*	0.284848485	NA	-0.563636364	NA
	Salinity	-0.76969697	-0.77	0.418181818	NA
**Shannon**	Depth	-0.769344739	-0.77	0.420230319	NA
	Tem	0.684848485	0.68	-0.357575758	NA
	DO	-0.696969697	-0.7	0.090909091	NA
	Chl *a*	0.539393939	NA	-0.648484848	-0.65
	Salinity	-0.757575758	-0.76	0.406060606	NA

*Tem, temperature.

Supplementary figures:

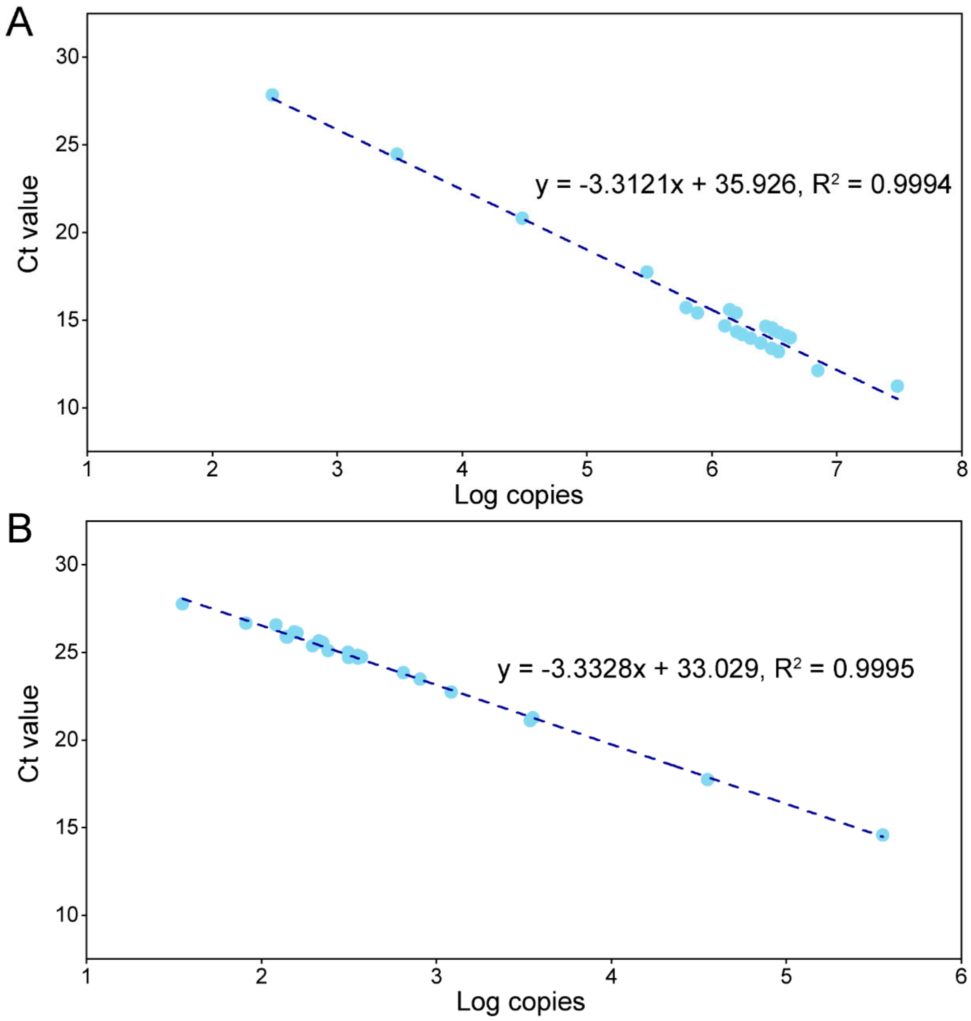

Figure S1 The standard curves of total bacteria (A) and *Vibrio* spp. (B) for qPCR quantification. Blue points mean each sample.

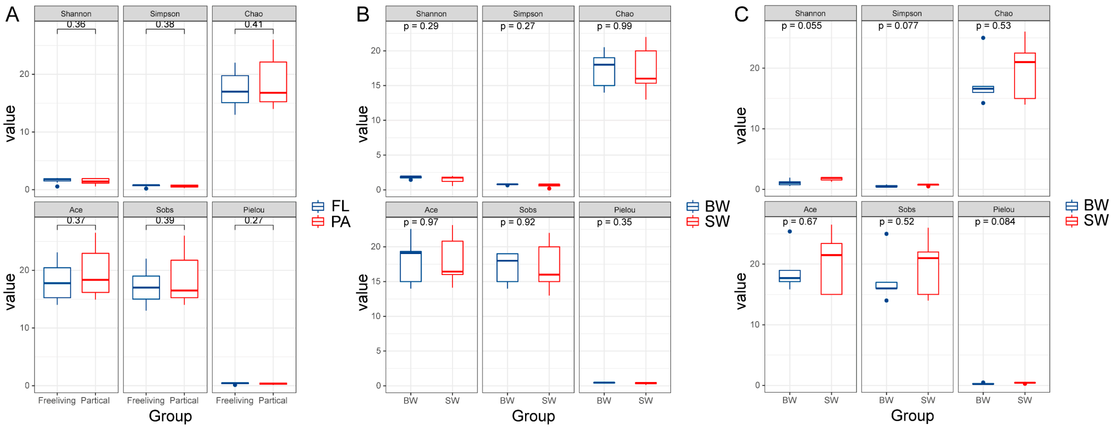

Figure S2 α-diversity indices of *Vibrio* community. The differences of values between FL and PA (A), BW and SW in FL (B) and PA (C) samples were calculated by student's *t*-test.

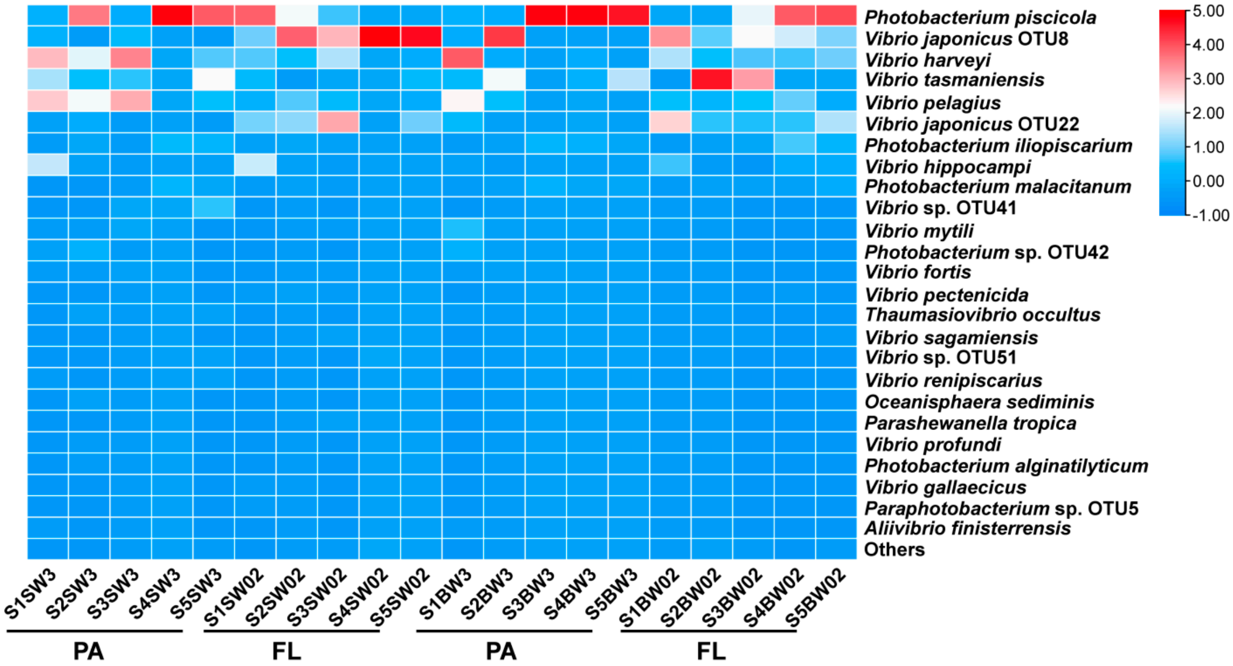

Figure S3 The heatmap for the relative abundance of *Vibrio* spp. in each sample. FL: free-living group, PA: particle-associated group.

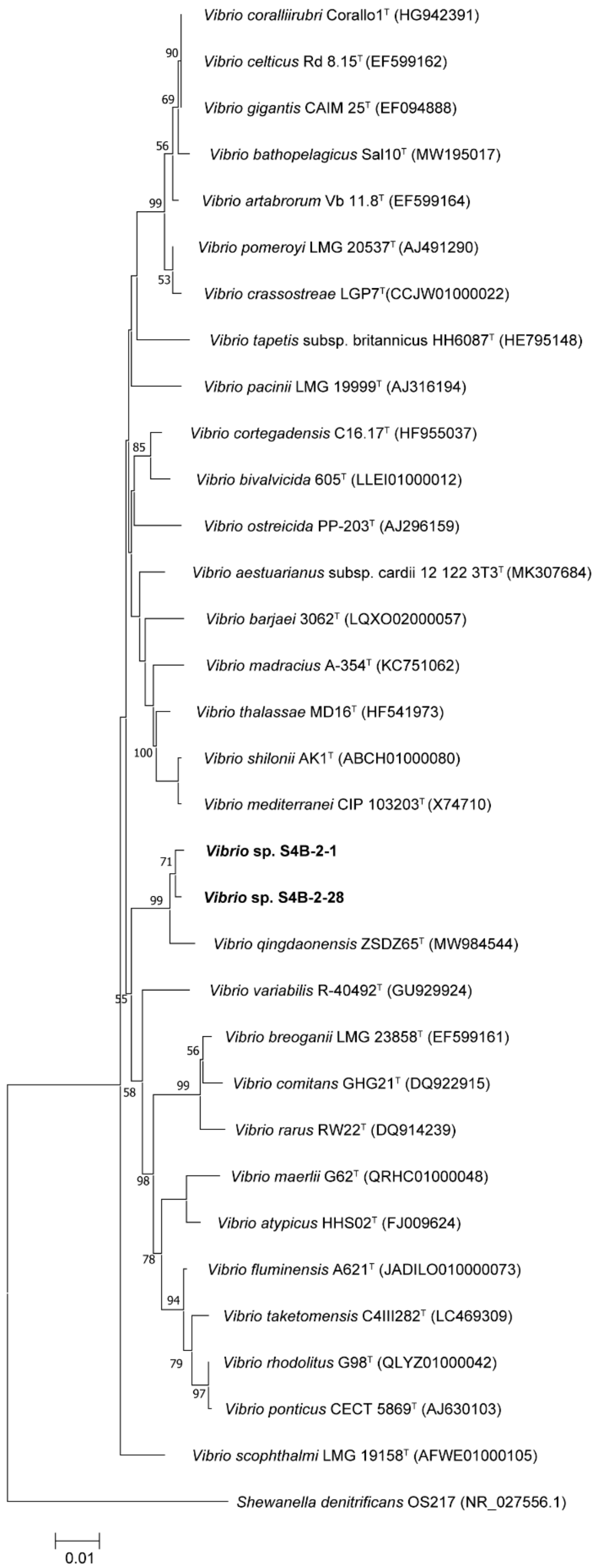

Figure S4 Neighbour-joining phylogenetic tree based on 16S rRNA gene sequences showing the phylogenetic position of potentially novel strains and other closely related species. Percentage bootstrap values above 50% (1000 replicates) are shown at branch nodes. Bar, 0.01 nucleotide substitutions per

nucleotide position.
